# RBFOX2: a key factor in suppressing PDAC metastasis through regulation of alternative splicing events

**DOI:** 10.1038/s41392-023-01585-3

**Published:** 2023-09-15

**Authors:** Laura Urbach, Shiv K. Singh

**Affiliations:** 1 Department of Gastroenterology, Gastrointestinal Oncology and Endocrinology, University Medical Center Göttingen, Göttingen, Germany; 2 Clinical Research Unit 5002, KFO5002, University Medical Center Göttingen, Göttingen, Germany

**Keywords:** Gastrointestinal cancer, Cancer

A new study published in *Nature* by Jbara et al. has identified the splicing factor “RBFOX2,” which halts metastatic spread of pancreatic ductal adenocarcinoma (PDAC) through regulating alternative splicing events.^1^ The results of the study provide new insights into the molecular mechanisms underlying the start of metastatic spread of PDAC cells. The study also indicates that pathways involved in splicing modulation could be targeted for potential therapeutic interventions in an advanced-stage PDAC.^1^

RNA splicing is a complex event, where introns are removed and exons are chained together to build a mature messenger RNA (mRNA). The alternative splicing occurs when certain exons are skipped, creating distinct mRNA isoforms. Spliceosomes are RNA-binding proteins that regulate alternative RNA splicing during cellular development. In cancer, dysregulation of spliceosome-mediated alternative splicing events has been found to associate with tumor progression and disease aggressiveness.^2,3^ It is interesting to note that while genetic mutations in splicing factors can cause oncogenic mis-splicing in various cancer types, coordinated splicing actually shapes cellular identity and prevents tumor progression and metastasis.^2^ Besides, oncogenic mutation in the *TP53* tumor suppressor gene can negatively regulate splicing factors in PDAC, leading to tumor progression and metastasis.^3^ Thus, cancer cells use alternative splicing to become more aggressive and maintain their survival.^2,3^ Whether and how alternative splicing events promote PDAC progression and metastasis, is not fully understood.

One-third of PDAC patients are diagnosed with local or distant metastasis, where chemotherapy with gemcitabine plus nab-paclitaxel and mFOLFIRINOX is the only potentially curative option; unfortunately, only a small cohort of patients respond to this treatment.^4,5^ Extensive molecular heterogeneity in the neoplasm and complex desmoplastic microenvironment facilitate PDAC aggressiveness.^5^ Thus, it warrants advancement in the molecular screening for early detection markers, and stratification-based therapy to improve the overall clinical outcome in PDAC patients.

Splicing factors present as promising therapeutic candidates since they shape mRNA composition and activity post-transcriptionally, thereby influencing the outcome of previous genetic or epigenetic events within the cells.^2,3^ In this study, the authors utilized publicly available RNA-sequencing (RNA-seq) datasets from 395 PDAC patient samples and stratified the read-out according to alternative splicing regulations. Furthermore, they separated the samples into two clusters that correlated with either primary tumors, covering histological stages I–III, or advanced stage IV.^5^ The study excluded samples that did not match clinical annotation to avoid bias, which could be averted. However, the findings were strengthened by follow-up experiments that showed a positive correlation between alternative splicing and metastatic spread in PDAC patients. Using de novo sequence motif analysis, the authors identified a significant enrichment of “RBFOX2” binding in various spliced regions, mainly in the primary tumors compared to metastatic PDAC. To identify the putative RBFXO2-bound regions, the authors used integrated transcriptome analysis where RHO GTPase pathway was found to be the main target of RBFOX2. Of note, the components of RHO GTPase pathway (RHOA, CDC42 and RAC1) are known regulators of focal adhesion and migration, indicating that loss of RBFOX2 expression could be linked to cellular motility. Indeed, the human PDX-derived metastatic PDAC cells showed loss of RBFOX2 expression compared to very high levels of RBFOX2 in primary PDAC cells. The authors showed that RBFOX2 overexpression significantly reduced the metastatic ability of human metastatic PDAC cells, while genetic silencing of RBFOX2 in primary PDAC cells accelerated metastases formation in preclinical models, suggesting that RBFOX2 is a metastatic suppressor in PDAC.^1^

**Fig. 1:**
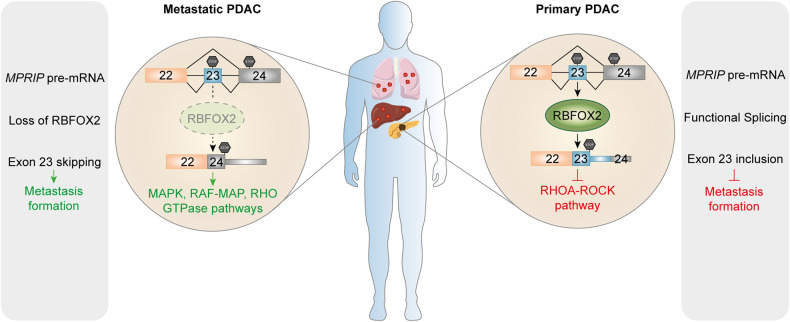
The role of RBFOX2-mediated alternative splicing in pancreatic cancer metastasis. The figure shows a schematic overview of the main findings made by Jbara et al.^1^ In the primary PDAC tumor, RBFOX2 abundance is high and assures correct splicing of targets such as MPRIP pre-mRNA. The final isoform includes all three exons 22, 23, and 24 forming a functional MPRIP protein that is involved in the inhibition of the RHOA-ROCK pathway and thus also metastasis formation. In the metastatic tumor specimen, however, RBFOX2 abundance is very low leading to mis-splicing of the RBFOX2 target MPRIP and eventually to the expression of an alternative protein isoform of MPRIP where exon 23 is skipped. This oncogenic isoform participates in the MAPK, RAF-MAP and RHO GTPase pathways thereby mediating metastasis formation. Some figure elements in this work were adapted from our previous publication^4^

While exploring the mechanisms of alternative splicing events, they identified that RBFOX2 directly controlled splicing of *MPRIP*, *MYL6*, and *CLSTN1* genes, which appeared to be critical for PDAC progression and metastases. For instance, RBFOX2 overexpression increased the expression of MPRIP longer isoform (i.e., inclusion of exon 23), whereas RBFOX2 silencing generated a shorter isoform (i.e., skipping of exon 23) in PDAC cells (Fig. 1). This study elegantly shows that re-expression of the shorter isoforms of MPRIP, MYL6, and CLSTN1 in primary PDAC cells substantially enhances metastatic spread in the liver and lung. To identify the mechanism of action, AlphaFold structure and serine-threonine kinome analyses were performed to predict the possible phosphorylation site on the MPRIP skipped exon 23. Finally, they discovered that the C-terminus of the skipped exon 23 had an α-helical structure, and it has various phosphorylation sites in each MPRIP splicing isoform. Notably, this skipped isoform of MPRIP was identified as a MAPK and RHO GTPase pathway interactor and was highly abundant in metastatic PDAC, accompanied by worse survival of PDAC patients.^1^ This highlights the importance of a functional splicing machinery in preventing metastatic spread since only one mis-spliced protein can have tremendous effects on the tumor integrity.^1^ As RBFOX2^loss^-MPRIP^skip^–RHO axis is critical for metastatic spread, the authors employed two distinct pharmacological inhibitors such as MBQ167 and azathioprine against RHO GTPase pathways. Importantly, both inhibitors drastically slowed down migration and metastatic spread of PDAC cells in preclinical mouse models, which extends the relevance of the study to promising targeted therapies for advanced stage PDAC. However, these two inhibitors had no considerable impact on proliferation and primary tumor growth, indicating that RBFOX2^loss^-MPRIP^skip^ regulatory axis exclusively controls the invasive metastatic route through RHO GTPase pathways.^1^

Increased oncogenic *KRAS* copy number and p53 mutation are substantially linked to high incidence of metastasis and therapy resistance profile in PDAC patients.^3,4^ A recent study showed that the mutant p53 promotes *KRAS* activity by regulating splicing factors, leading to PDAC progression and metastasis.^3^ In this study, it was yet to be determined which factors, whether oncogenic or not, control RBFOX2 during PDAC progression and metastatic spread. Of clinical interest, the extent to which the expression of RBFOX2 and MPRIP skipping events affect first-line therapy response and facilitate tumor relapse in PDAC patients remains to be examined. Further studies addressing whether RBFOX2-mediated alternative splicing events associate with genomic alterations, basal-like/squamous or classical transcriptome subtype, as well as modulating stromal immune microenvironment will not only lead to a better understanding of complex PDAC heterogeneity, but will also offer early prognosis and precision-based therapeutic development.

In summary, Jbara and colleagues have explored an interesting new approach to stratify PDAC patients according to their alternative splicing landscape in disease aggressiveness.^1^ This adds another layer to previous perspectives focusing on transcriptional and genetic alterations and has potential to expand these findings for a better understanding of PDAC plasticity and progression. Altogether, extrapolating the insightful knowledge obtained via this study about splicing factors, splicing isoforms and their potential to control disease aggressiveness, early metastatic spread and therapy resistance, could pave way to understanding comprehensively the molecular basis of PDAC pathogenesis.
